# Supplementary material for: Inferring Dynamic Information from Protein Structures by Gaussian Integrals and Deep Learning
Source: bioRxiv. 2025 Sep 24:2025.09.22.677755. Preprint. [Version 1] doi: 10.1101/2025.09.22.677755 (PMC12485704; doi:10.1101/2025.09.22.677755)
Supplement: Supplement 1 [file NIHPP2025.09.22.677755v1-supplement-1.pdf]

bioRxiv preprint doi: https://doi.org/10.1101/2025.09.22.677755; this version posted September 24, 2025. The copyright holder for this preprint (which was not certified by peer review) is the author/funder, who has granted bioRxiv a license to display the preprint in perpetuity. It is made available under aCC-BY 4.0 International license.

Supplementary Figure 1. Additional analyses for the classification task. **A)** Predicted probability distributions across clusters for proteins labeled as non-flexible (class 0) and flexible (class 1). **B)** Training and validation binary cross-entropy loss curves across epochs. **C)** Feature attribution analysis using Integrated Gradients showing the relative contribution of each GI position (1–30) to the classification model.

Supplementary Figure 2. Additional analyses for the regression task. **A)** Zoomed scatter plot of predicted versus true RMSF values for proteins with RMSF ≤ 5Angstrom. **B)** Training and

bioRxiv preprint doi: https://doi.org/10.1101/2025.09.22.677755; this version posted September 24, 2025. The copyright holder for this preprint (which was not certified by peer review) is the author/funder, who has granted bioRxiv a license to display the preprint in perpetuity. It is made available under aCC-BY 4.0 International license.

validation loss curves across epochs for the regression model, with shaded areas indicating standard deviation.

bioRxiv preprint doi: https://doi.org/10.1101/2025.09.22.677755; this version posted September 24, 2025. The copyright holder for this preprint (which was not certified by peer review) is the author/funder, who has granted bioRxiv a license to display the preprint in perpetuity. It is made available under aCC-BY 4.0 International license.

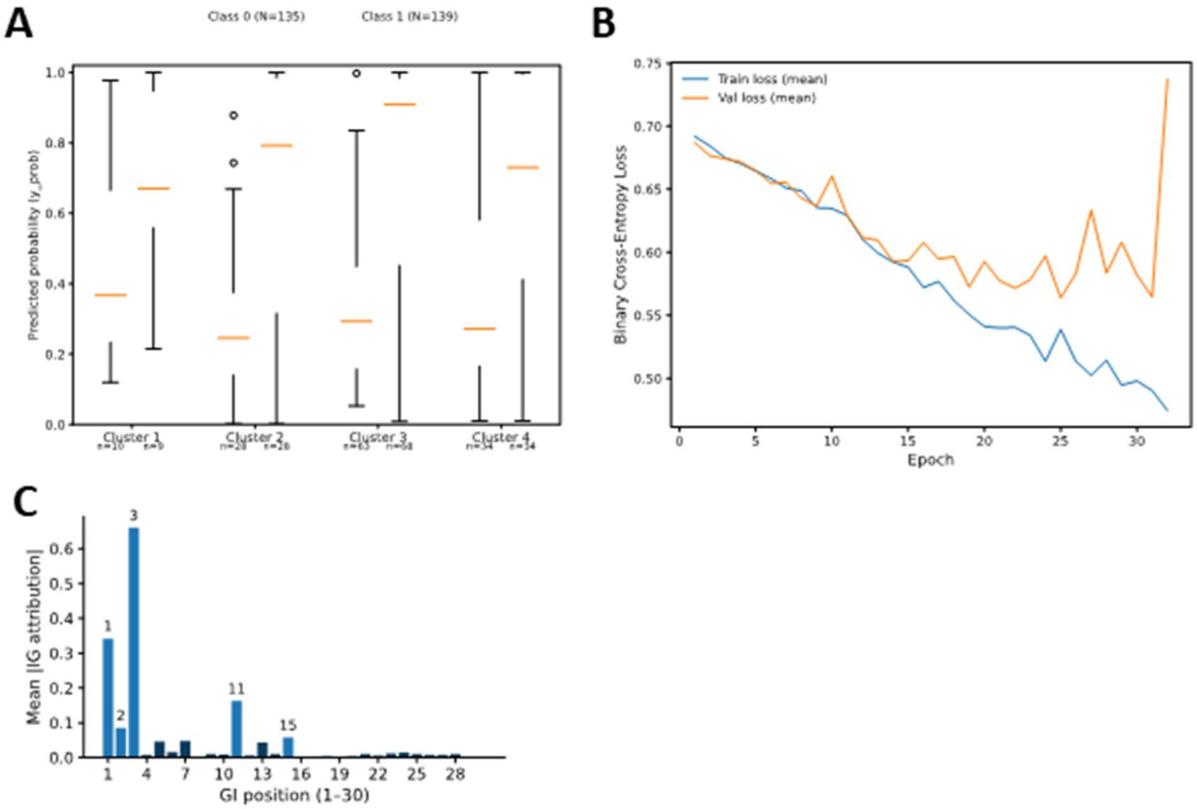

Figure S1

bioRxiv preprint doi: https://doi.org/10.1101/2025.09.22.677755; this version posted September 24, 2025. The copyright holder for this preprint (which was not certified by peer review) is the author/funder, who has granted bioRxiv a license to display the preprint in perpetuity. It is made available under aCC-BY 4.0 International license.

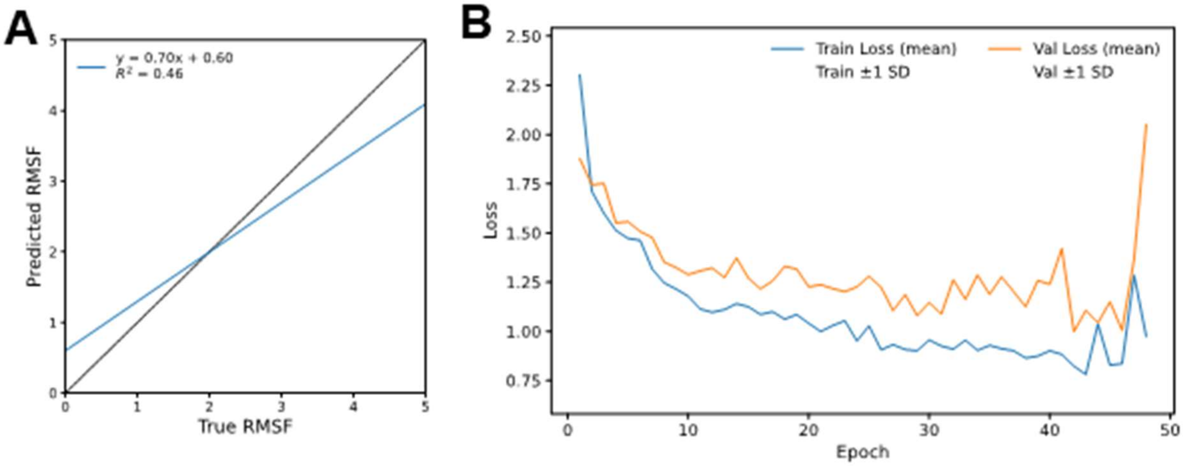

Figure S2
